# Supplementary material for: Ligand-Independent Vitamin D Receptor Actions Essential for Keratinocyte Homeostasis in the Skin
Source: Int J Mol Sci. 2025 Jan 6;26(1):422. doi: 10.3390/ijms26010422 (PMC11720424; doi:10.3390/ijms26010422)
Supplement: Supplementary file 1 [file ijms-26-00422-s001.zip › ijms-3342575-supplementary.pptx]

## Slide 1
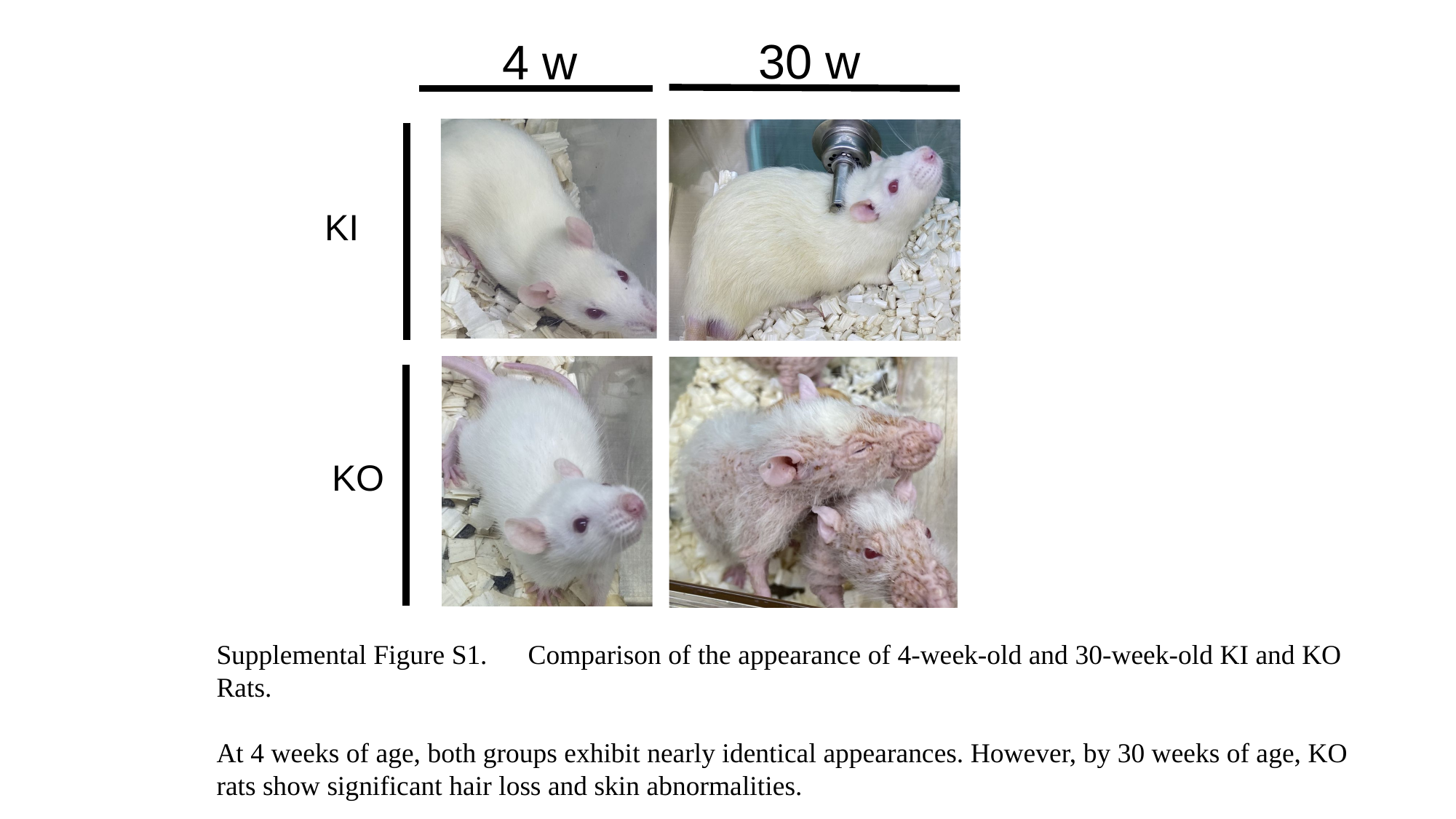

30 w
4 w
KI
KO
Supplemental Figure S1.　Comparison of the appearance of 4-week-old and 30-week-old KI and KO Rats.
At 4 weeks of age, both groups exhibit nearly identical appearances. However, by 30 weeks of age, KO rats show significant hair loss and skin abnormalities.

## Slide 2
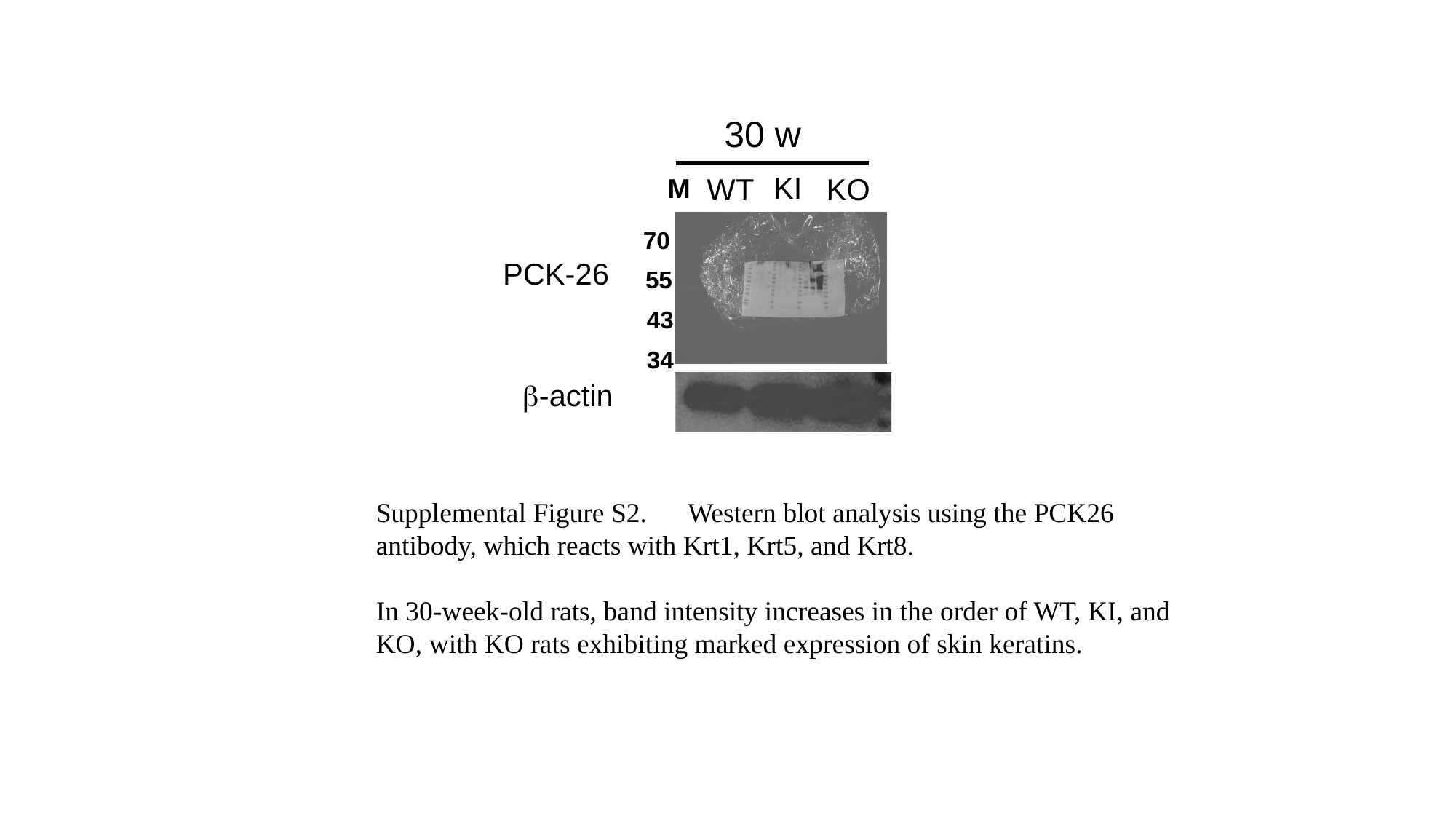

30 w
KI
KO
WT
M
70
PCK-26
55
43
34
b-actin
Supplemental Figure S2.　Western blot analysis using the PCK26 antibody, which reacts with Krt1, Krt5, and Krt8.
In 30-week-old rats, band intensity increases in the order of WT, KI, and KO, with KO rats exhibiting marked expression of skin keratins.

## Slide 3
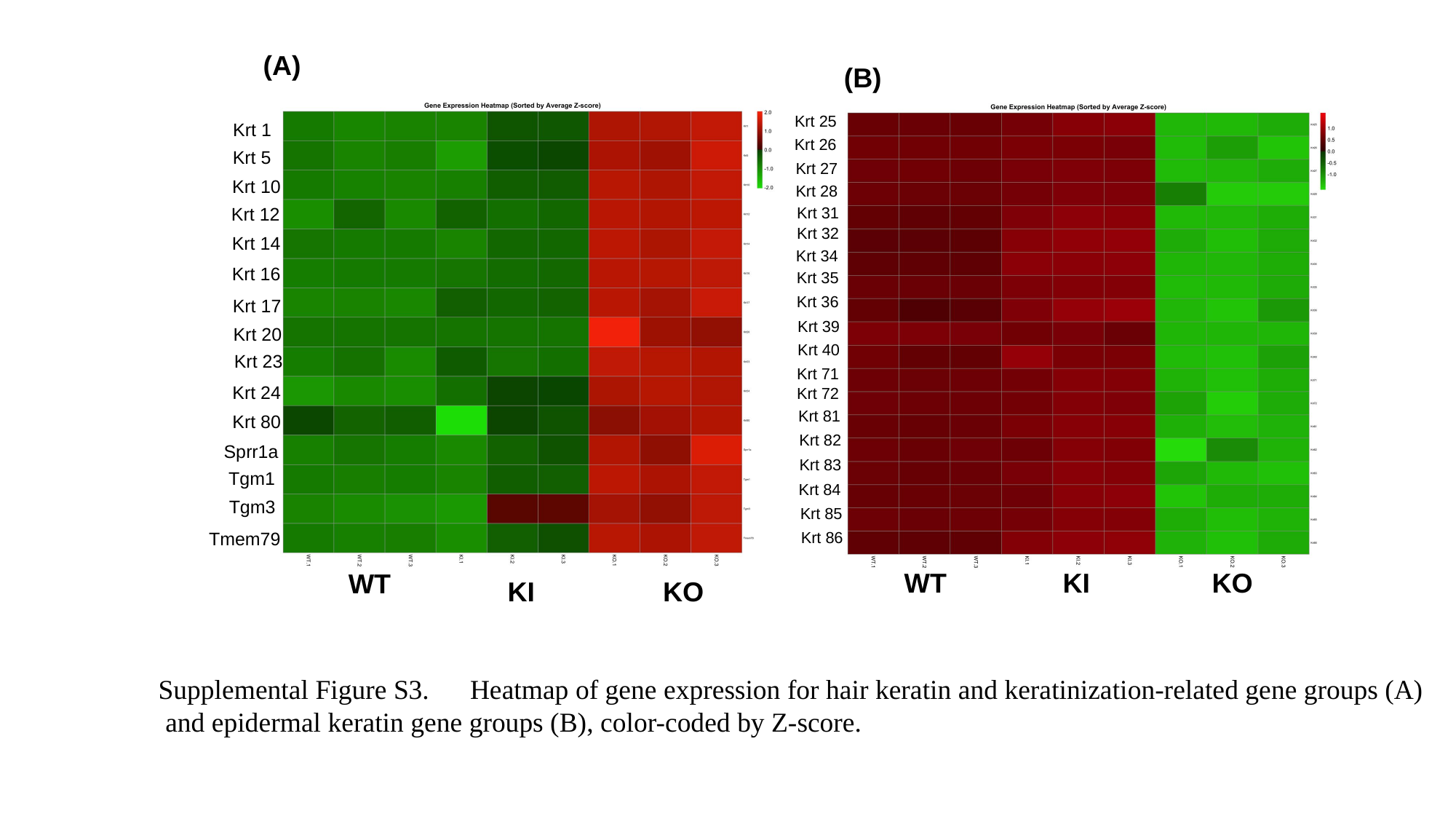

(A)
(B)
Krt 25
Krt 1
Krt 26
Krt 5
Krt 27
Krt 10
Krt 28
Krt 31
Krt 12
Krt 32
Krt 14
Krt 34
Krt 16
Krt 35
Krt 36
Krt 17
Krt 39
Krt 20
Krt 40
Krt 23
Krt 71
Krt 24
Krt 72
Krt 81
Krt 80
Krt 82
Sprr1a
Krt 83
Tgm1
Krt 84
Tgm3
Krt 85
Krt 86
Tmem79
KI
WT
KO
WT
KI
KO
Supplemental Figure S3.　Heatmap of gene expression for hair keratin and keratinization-related gene groups (A)
 and epidermal keratin gene groups (B), color-coded by Z-score.

## Slide 4
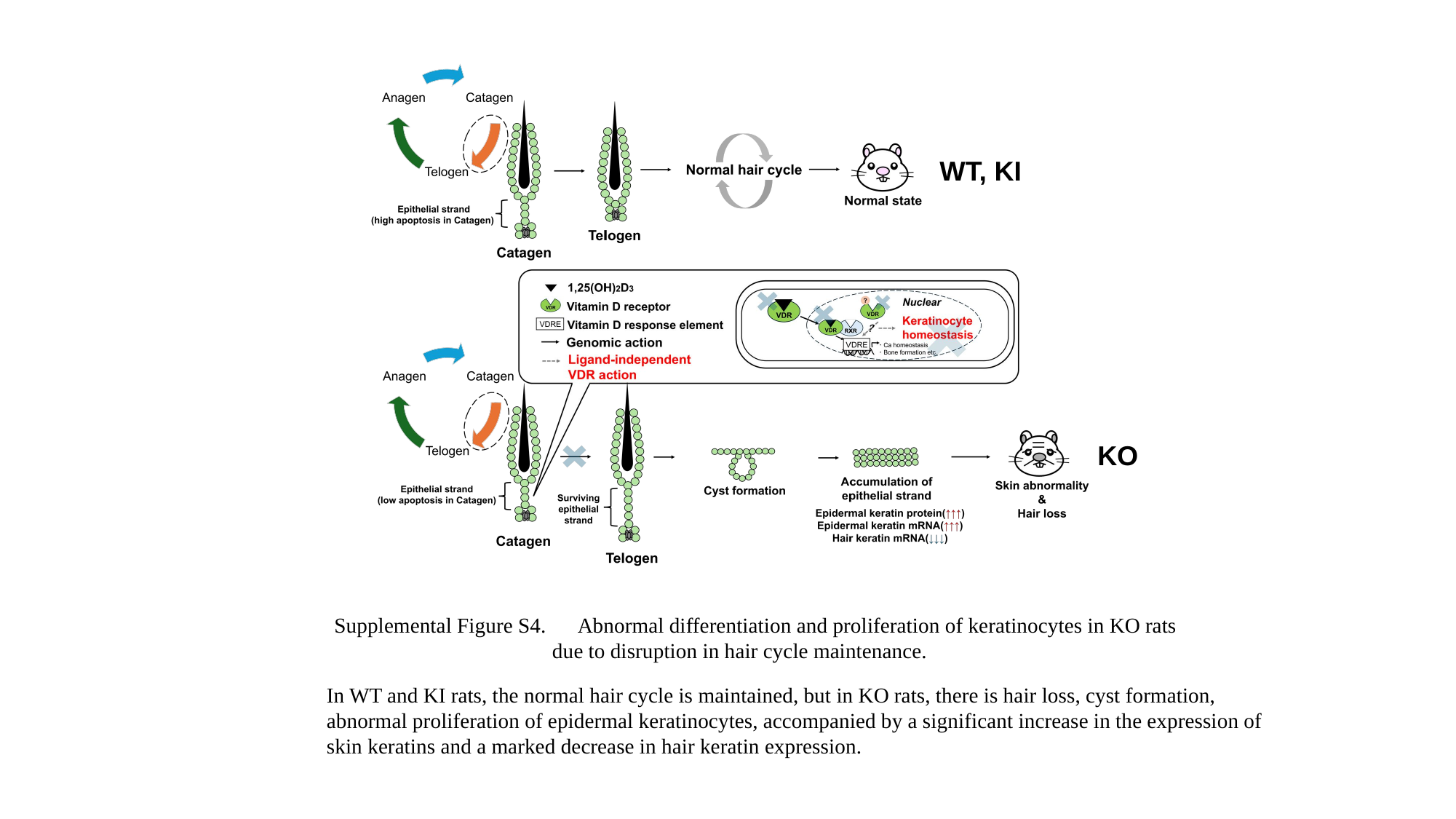

WT, KI
KO
Supplemental Figure S4.　Abnormal differentiation and proliferation of keratinocytes in KO rats
　　　　　　　　　　due to disruption in hair cycle maintenance.
In WT and KI rats, the normal hair cycle is maintained, but in KO rats, there is hair loss, cyst formation,
abnormal proliferation of epidermal keratinocytes, accompanied by a significant increase in the expression of skin keratins and a marked decrease in hair keratin expression.

## Slide 5
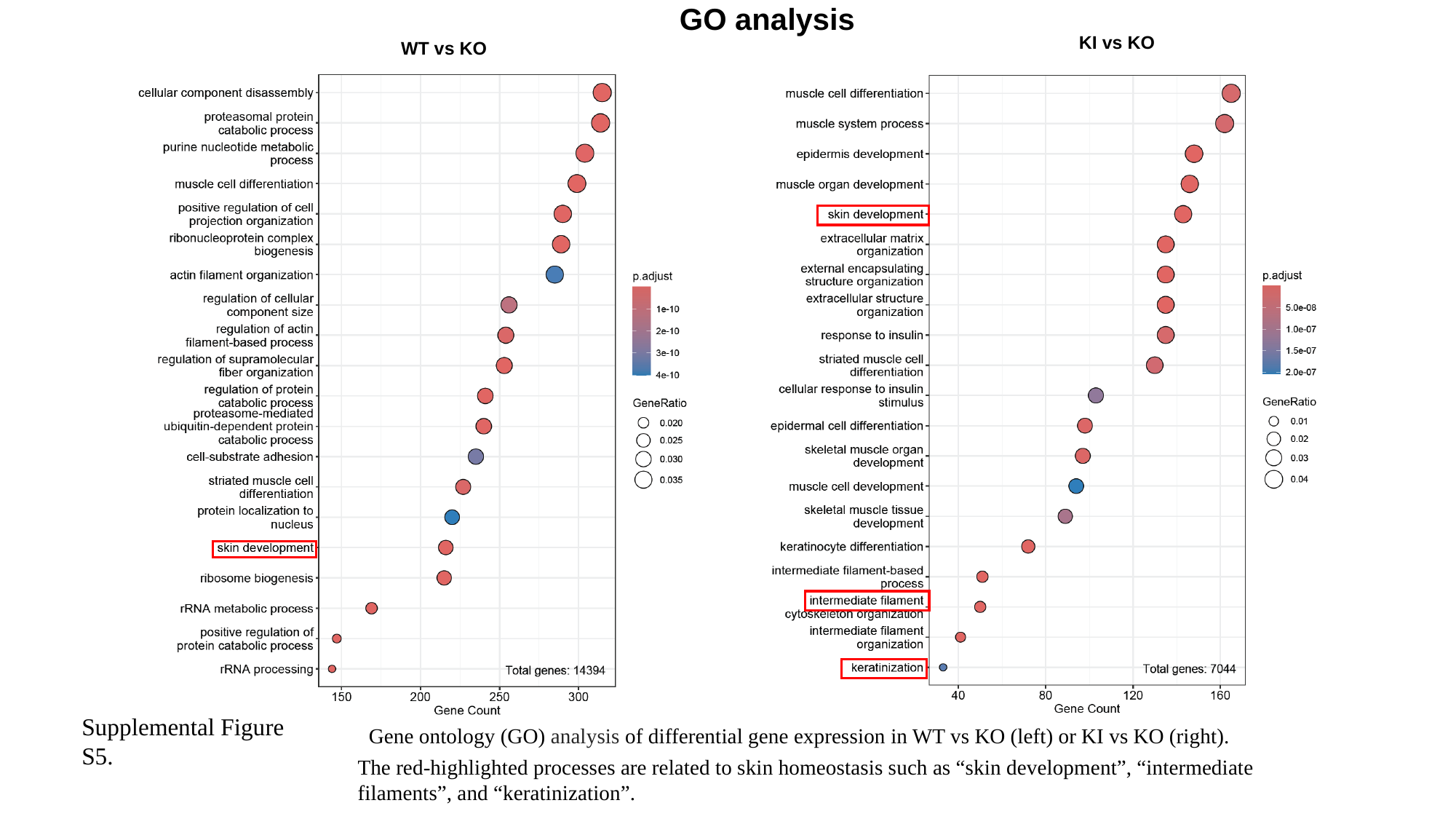

GO analysis
KI vs KO
WT vs KO
Supplemental Figure S5.
Gene ontology (GO) analysis of differential gene expression in WT vs KO (left) or KI vs KO (right).
The red-highlighted processes are related to skin homeostasis such as “skin development”, “intermediate filaments”, and “keratinization”.

## Slide 6
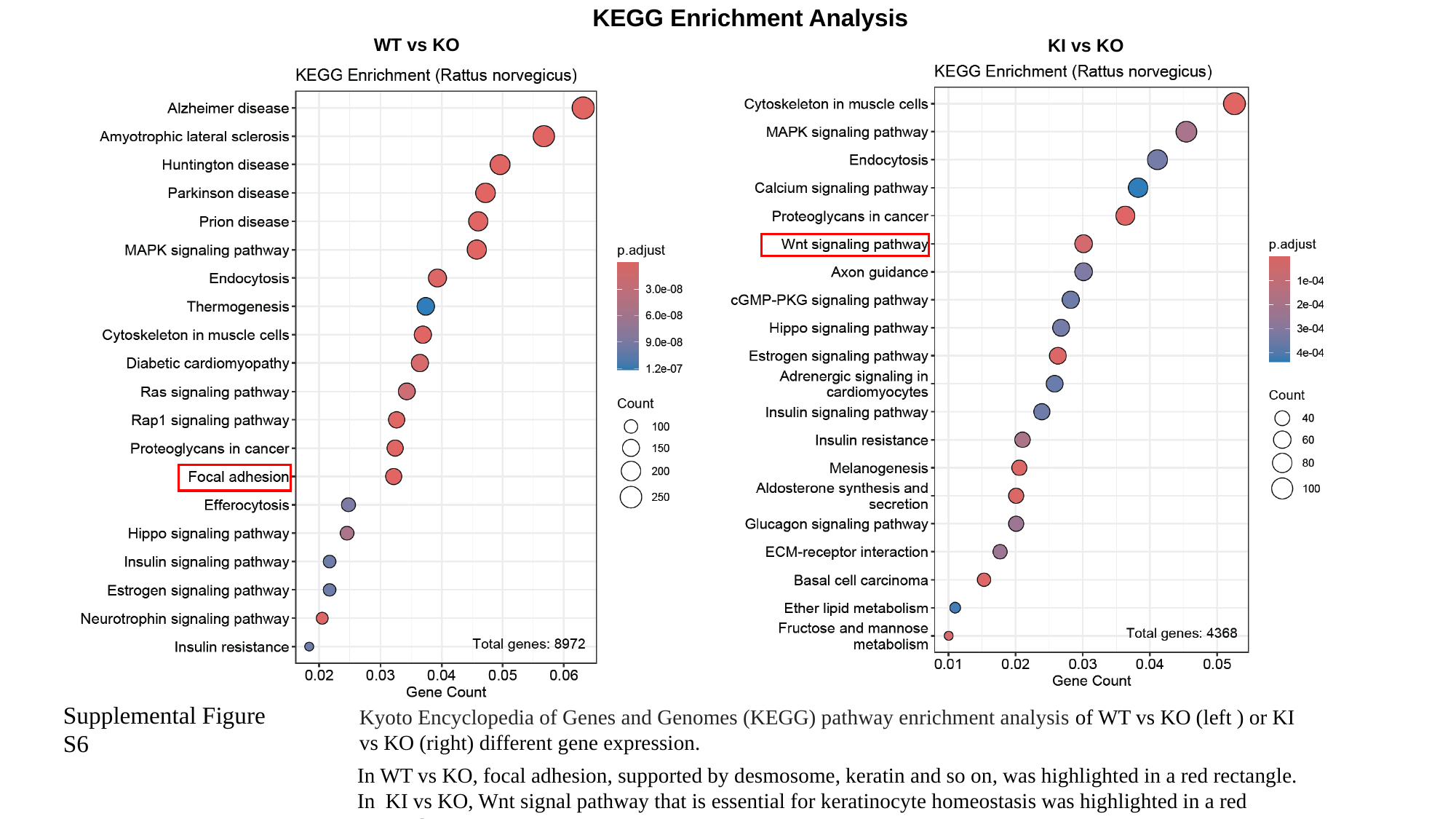

KEGG Enrichment Analysis
WT vs KO
KI vs KO
Supplemental Figure S6
Kyoto Encyclopedia of Genes and Genomes (KEGG) pathway enrichment analysis of WT vs KO (left ) or KI vs KO (right) different gene expression.
In WT vs KO, focal adhesion, supported by desmosome, keratin and so on, was highlighted in a red rectangle.
In KI vs KO, Wnt signal pathway that is essential for keratinocyte homeostasis was highlighted in a red rectangle.

## Slide 7
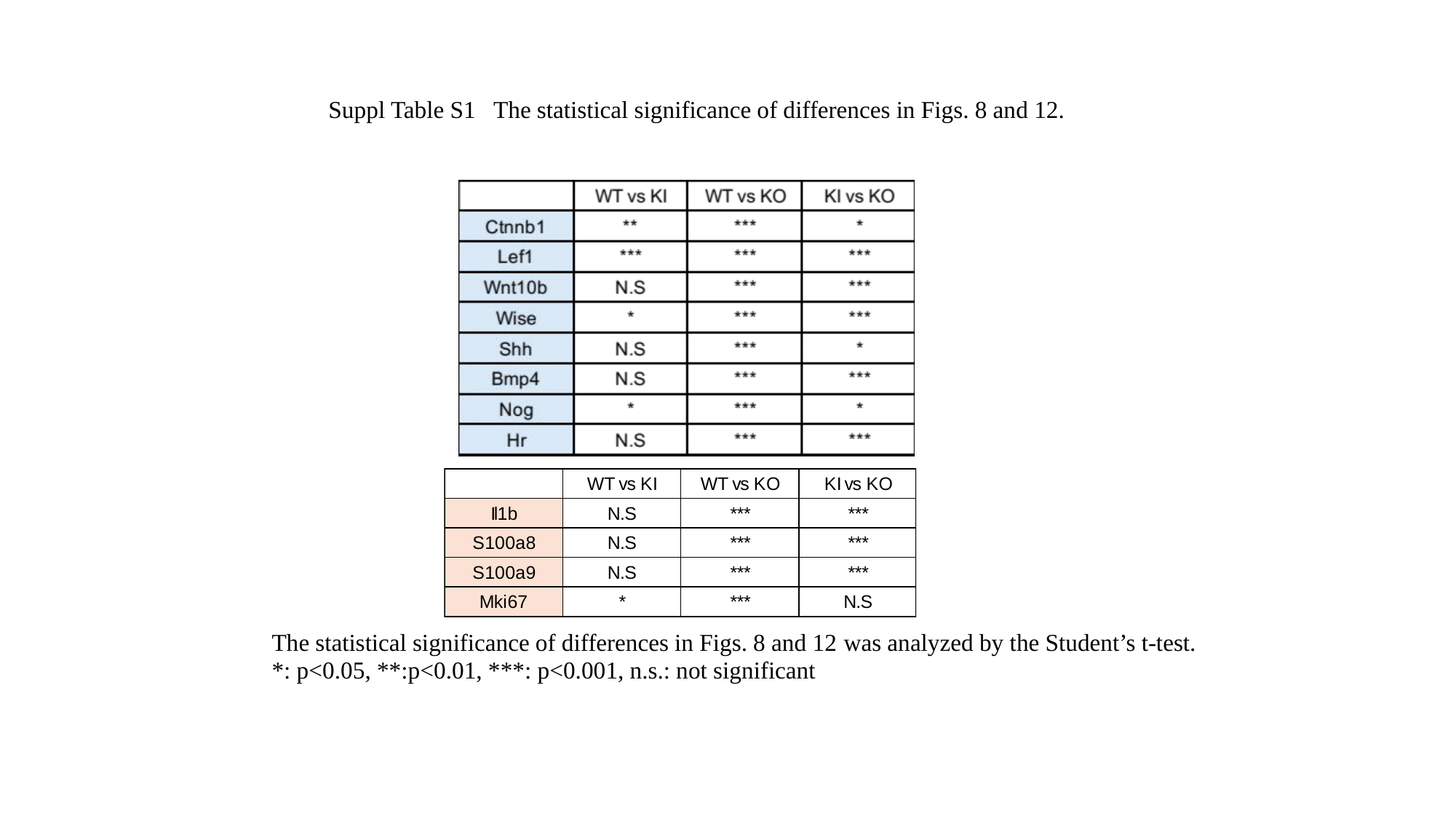

Suppl Table S1 The statistical significance of differences in Figs. 8 and 12.
The statistical significance of differences in Figs. 8 and 12 was analyzed by the Student’s t-test. *: p<0.05, **:p<0.01, ***: p<0.001, n.s.: not significant

## Slide 8
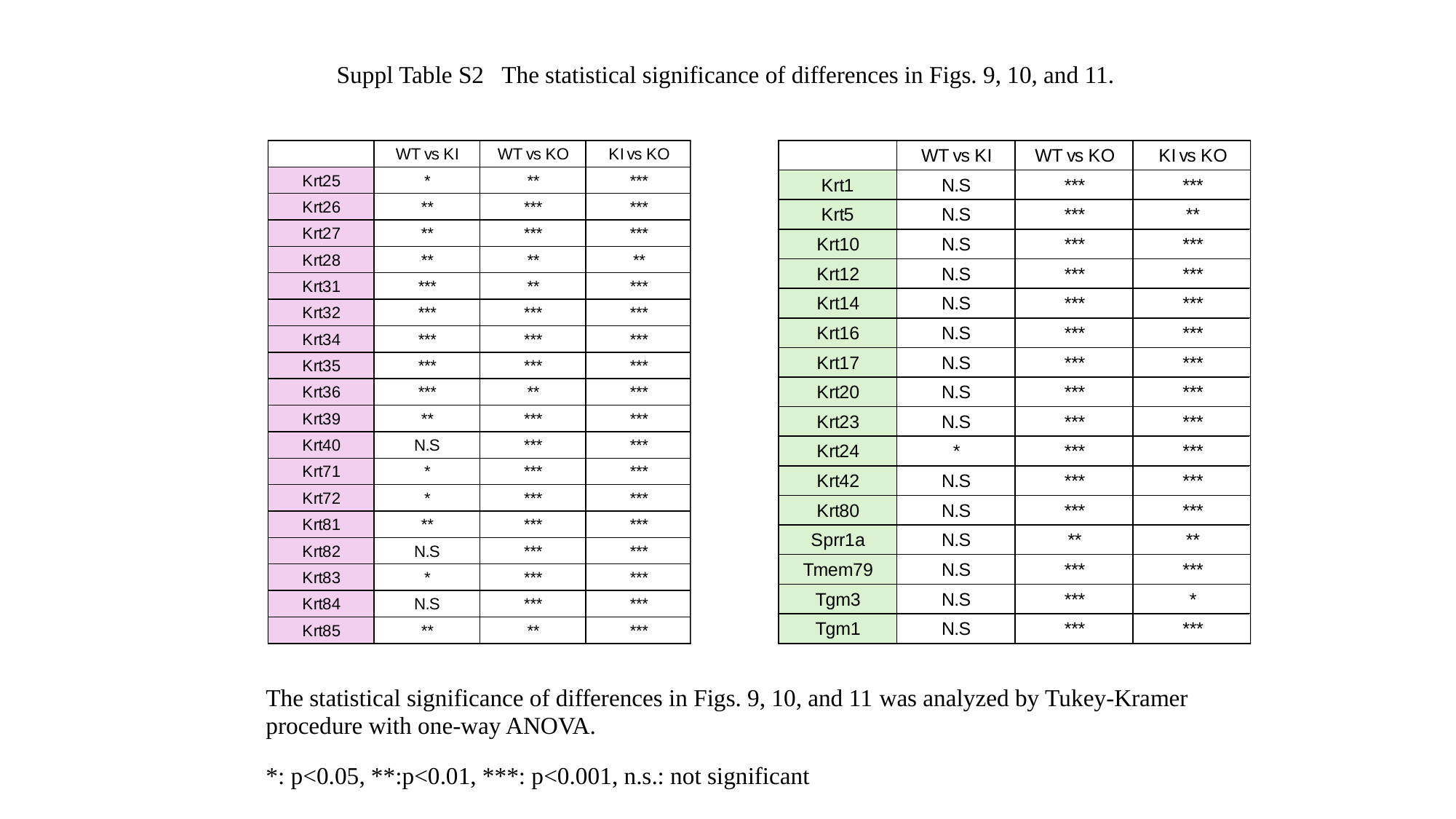

Suppl Table S2 The statistical significance of differences in Figs. 9, 10, and 11.
The statistical significance of differences in Figs. 9, 10, and 11 was analyzed by Tukey-Kramer procedure with one-way ANOVA.
*: p<0.05, **:p<0.01, ***: p<0.001, n.s.: not significant

## Slide 9
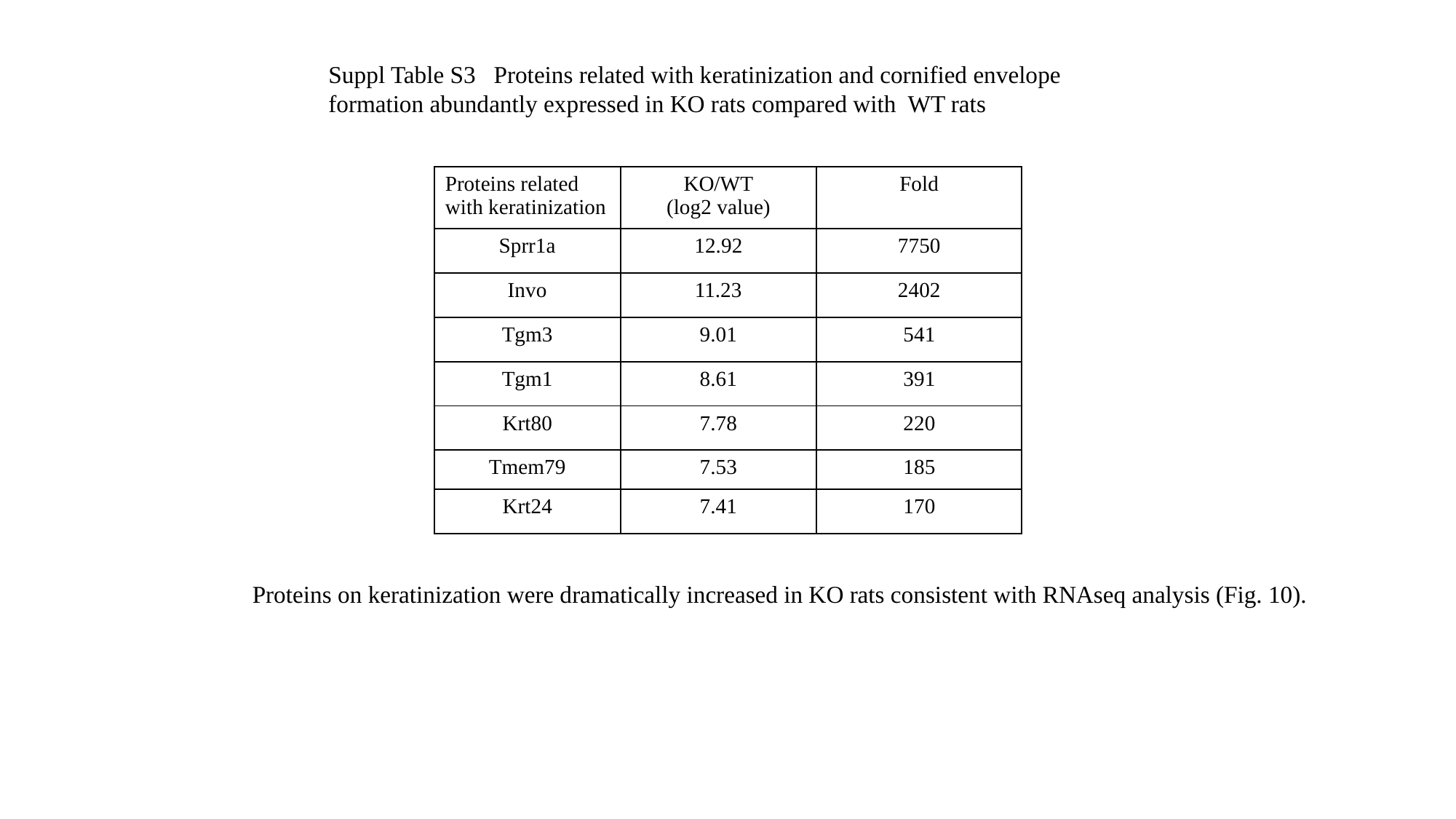

Suppl Table S3 Proteins related with keratinization and cornified envelope formation abundantly expressed in KO rats compared with WT rats
| Proteins related with keratinization | KO/WT (log2 value) | Fold |
| --- | --- | --- |
| Sprr1a | 12.92 | 7750 |
| Invo | 11.23 | 2402 |
| Tgm3 | 9.01 | 541 |
| Tgm1 | 8.61 | 391 |
| Krt80 | 7.78 | 220 |
| Tmem79 | 7.53 | 185 |
| Krt24 | 7.41 | 170 |
Proteins on keratinization were dramatically increased in KO rats consistent with RNAseq analysis (Fig. 10).

## Slide 10
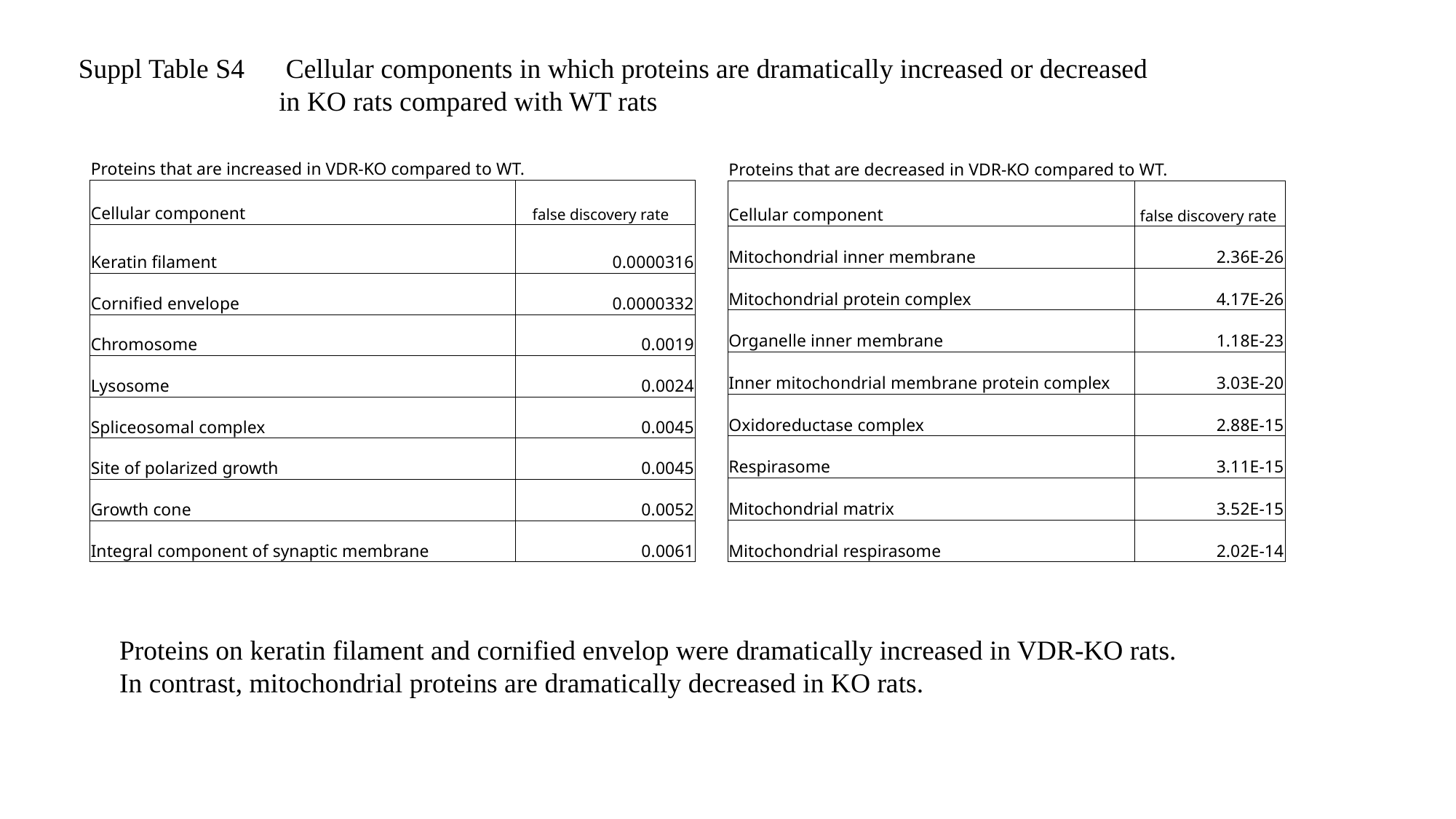

Suppl Table S4 Cellular components in which proteins are dramatically increased or decreased
 in KO rats compared with WT rats
| Proteins that are increased in VDR-KO compared to WT. | |
| --- | --- |
| Cellular component | false discovery rate |
| Keratin filament | 0.0000316 |
| Cornified envelope | 0.0000332 |
| Chromosome | 0.0019 |
| Lysosome | 0.0024 |
| Spliceosomal complex | 0.0045 |
| Site of polarized growth | 0.0045 |
| Growth cone | 0.0052 |
| Integral component of synaptic membrane | 0.0061 |
| Proteins that are decreased in VDR-KO compared to WT. | |
| --- | --- |
| Cellular component | false discovery rate |
| Mitochondrial inner membrane | 2.36E-26 |
| Mitochondrial protein complex | 4.17E-26 |
| Organelle inner membrane | 1.18E-23 |
| Inner mitochondrial membrane protein complex | 3.03E-20 |
| Oxidoreductase complex | 2.88E-15 |
| Respirasome | 3.11E-15 |
| Mitochondrial matrix | 3.52E-15 |
| Mitochondrial respirasome | 2.02E-14 |
Proteins on keratin filament and cornified envelop were dramatically increased in VDR-KO rats.
In contrast, mitochondrial proteins are dramatically decreased in KO rats.

## Slide 11
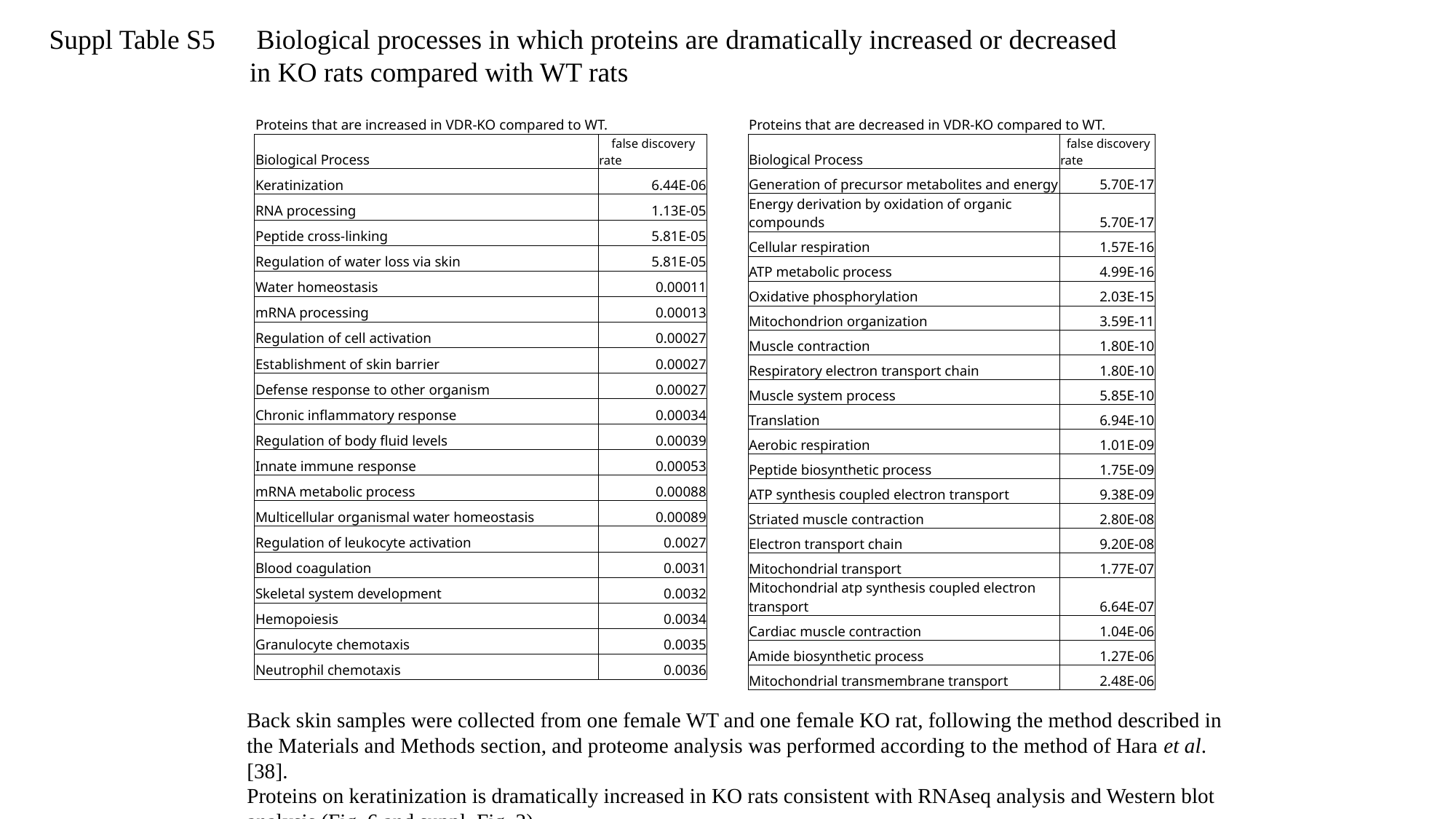

Suppl Table S5 Biological processes in which proteins are dramatically increased or decreased
 in KO rats compared with WT rats
| Proteins that are increased in VDR-KO compared to WT. | |
| --- | --- |
| Biological Process | false discovery rate |
| Keratinization | 6.44E-06 |
| RNA processing | 1.13E-05 |
| Peptide cross-linking | 5.81E-05 |
| Regulation of water loss via skin | 5.81E-05 |
| Water homeostasis | 0.00011 |
| mRNA processing | 0.00013 |
| Regulation of cell activation | 0.00027 |
| Establishment of skin barrier | 0.00027 |
| Defense response to other organism | 0.00027 |
| Chronic inflammatory response | 0.00034 |
| Regulation of body fluid levels | 0.00039 |
| Innate immune response | 0.00053 |
| mRNA metabolic process | 0.00088 |
| Multicellular organismal water homeostasis | 0.00089 |
| Regulation of leukocyte activation | 0.0027 |
| Blood coagulation | 0.0031 |
| Skeletal system development | 0.0032 |
| Hemopoiesis | 0.0034 |
| Granulocyte chemotaxis | 0.0035 |
| Neutrophil chemotaxis | 0.0036 |
| Proteins that are decreased in VDR-KO compared to WT. | |
| --- | --- |
| Biological Process | false discovery rate |
| Generation of precursor metabolites and energy | 5.70E-17 |
| Energy derivation by oxidation of organic compounds | 5.70E-17 |
| Cellular respiration | 1.57E-16 |
| ATP metabolic process | 4.99E-16 |
| Oxidative phosphorylation | 2.03E-15 |
| Mitochondrion organization | 3.59E-11 |
| Muscle contraction | 1.80E-10 |
| Respiratory electron transport chain | 1.80E-10 |
| Muscle system process | 5.85E-10 |
| Translation | 6.94E-10 |
| Aerobic respiration | 1.01E-09 |
| Peptide biosynthetic process | 1.75E-09 |
| ATP synthesis coupled electron transport | 9.38E-09 |
| Striated muscle contraction | 2.80E-08 |
| Electron transport chain | 9.20E-08 |
| Mitochondrial transport | 1.77E-07 |
| Mitochondrial atp synthesis coupled electron transport | 6.64E-07 |
| Cardiac muscle contraction | 1.04E-06 |
| Amide biosynthetic process | 1.27E-06 |
| Mitochondrial transmembrane transport | 2.48E-06 |
Back skin samples were collected from one female WT and one female KO rat, following the method described in the Materials and Methods section, and proteome analysis was performed according to the method of Hara et al. [38].
Proteins on keratinization is dramatically increased in KO rats consistent with RNAseq analysis and Western blot analysis (Fig. 6 and suppl. Fig. 2)
